# Supplementary figures and images for: Rapid and non-invasive detection of malaria parasites using near-infrared spectroscopy and machine learning
Source: PLoS One. 2024 Mar 25;19(3):e0289232. doi: 10.1371/journal.pone.0289232 (PMC10962802; doi:10.1371/journal.pone.0289232)

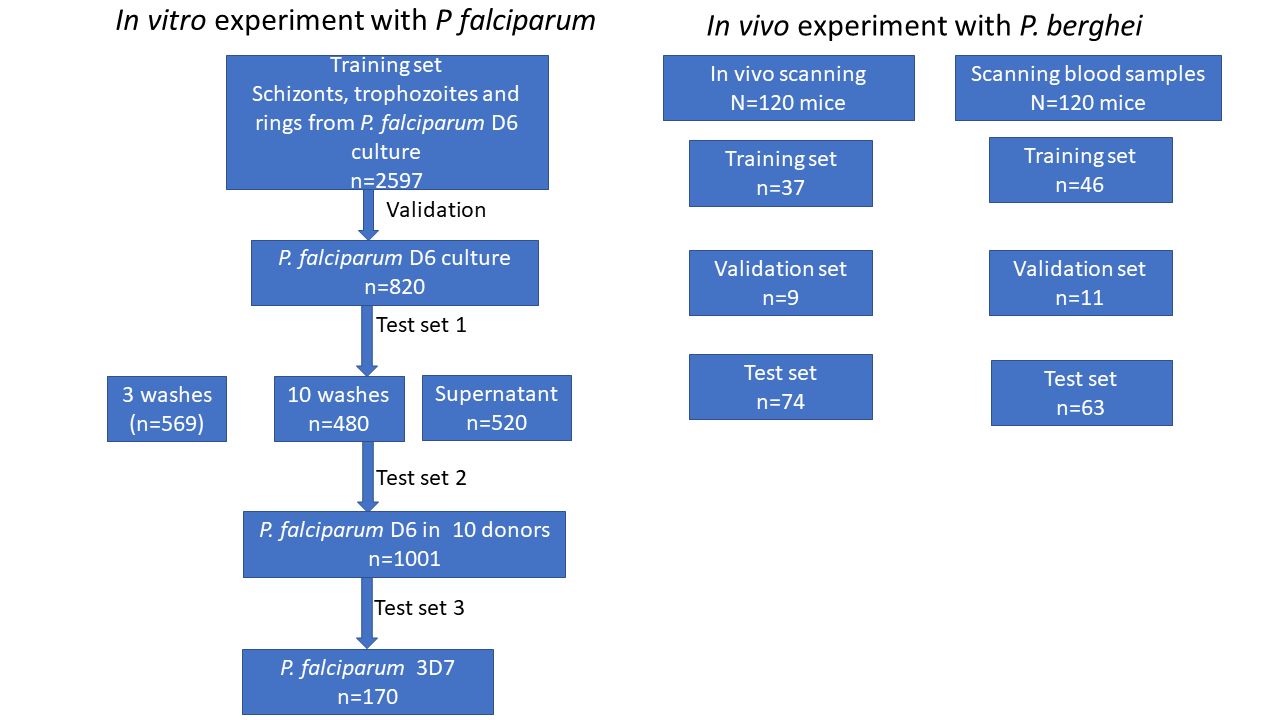

Supplement: S1 Fig — (TIF) [file pone.0289232.s001.tif]

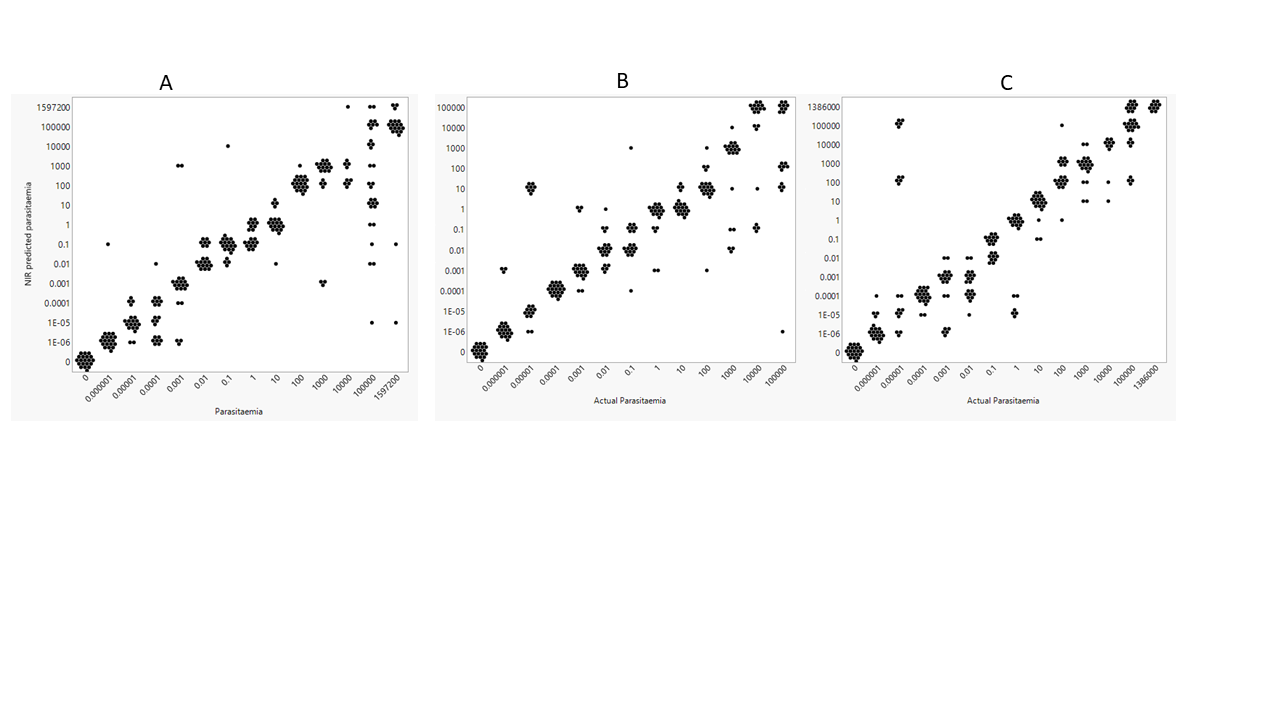

Supplement: S2 Fig — (TIF) [file pone.0289232.s002.tif]

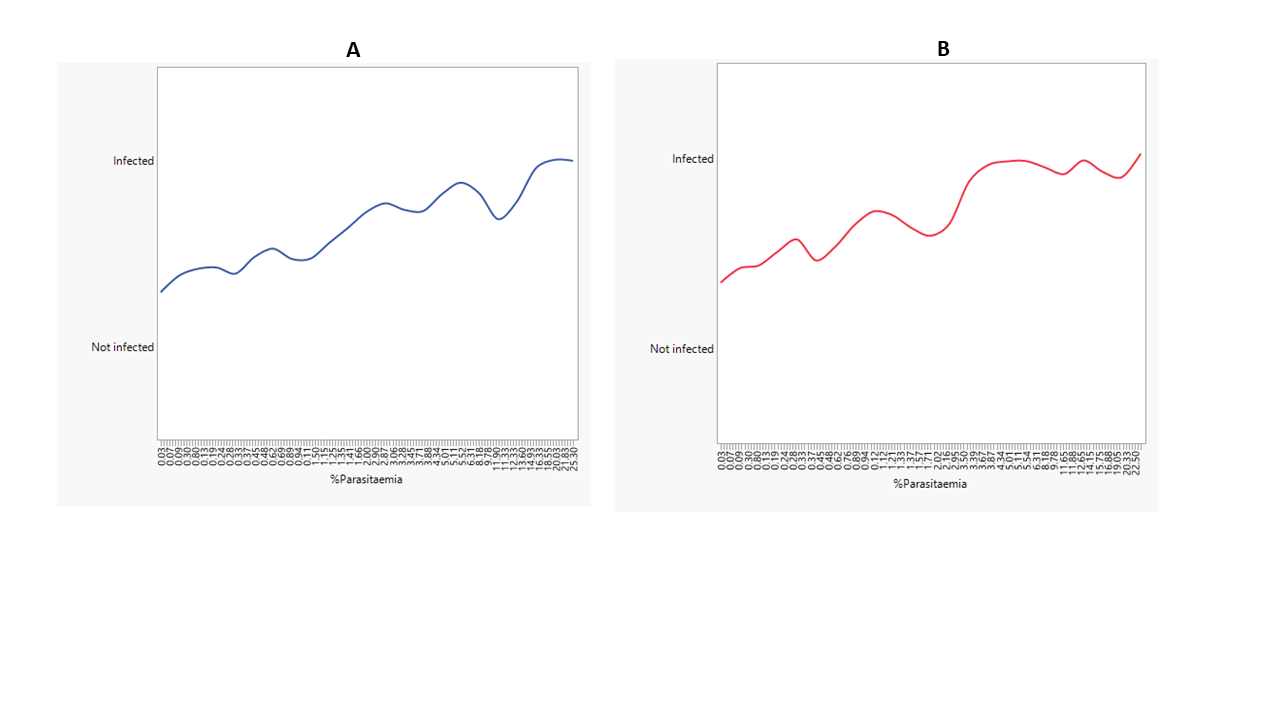

Supplement: S3 Fig — The effect of parasitemia for non-invasive (Panel A) and invasive (Panel B) prediction of P. berghei infection, where mice with high parasitemia levels are more likely to be predicted as infected compared to mice with lower parasitemia values. (TIF) [file pone.0289232.s003.tif]
