# Supplementary material for: Rapid and non-invasive detection of malaria parasites using near-infrared spectroscopy and machine learning
Source: PLoS One. 2024 Mar 25;19(3):e0289232. doi: 10.1371/journal.pone.0289232 (PMC10962802; doi:10.1371/journal.pone.0289232)
Supplement: Attachment — Submitted filename: Plos_Review.docx [file pone.0289232.s004.docx]

Title: Rapid and non-invasive detection of malaria parasites using near-infrared spectroscopy and machine learning

Manuscript is well written and data are well presented. Authors have addressed an interesting and essential aspect which needs to be addressed. The need for a non-invasive methodology is necessary in malaria endemic regions for quick screening of infected and uninfected subjects. The study has pointed out that NIS can screen the parasite accurately in limited time. Using different experimental setup, the authors have also shown the efficiency of this technique. I find the article interesting and a step forward in rapid and robust screening parasite on large scale in short frame of time.

Comments:

1) If possible, the authors may include hemolyzed samples for efficiency of this methodology for detection.
2) Further, authors have shown that the higher parasitemia is detected more accurately *in vivo* system this suggests that the lower limit of detection *in vivo* and *invitro* is different. Authors may discuss the factors which may be responsible for this difference.
3) It would have been more interesting to see the detection of malaria parasite in the infected individuals without any processing. The parasitemia in individual usually not high except under chronic condition or in highly endemic regions.
4) Is this methodology specifically related to *Plasmodium falciparum* or to other malarial parasite?
5) Authors assert that “specific components in biological samples” be responsible for the spectra change if true would spectra of urine from infected mice samples may also show change in spectra?

6) Throughout the study O^+^ blood was used is the data. Does the detection accuracy similar in all other blood groups?

Authors may discuss above points throughout the manuscript. Overall, I recommend this article for publication.
