# Supplementary material for: Rapid and non-invasive detection of malaria parasites using near-infrared spectroscopy and machine learning
Source: PLoS One. 2024 Mar 25;19(3):e0289232. doi: 10.1371/journal.pone.0289232 (PMC10962802; doi:10.1371/journal.pone.0289232)
Supplement: Attachment — Submitted filename: Response to Reviewers.docx [file pone.0289232.s005.docx]

**Response to Reviewers’**

Dear academic editor,

Please find below our response to academic editor’s comments and comments from reviewers shown in below under each comment. The line numbers provided refer to the revised manuscript with tracked changes.

Journal requirements:

When submitting your revision, we need you to address these additional requirements.

1. Please ensure that your manuscript meets PLOS ONE's style requirements, including those for file naming. The PLOS ONE style templates can be found at

<https://journals.plos.org/plosone/s/file?id=wjVg/PLOSOne_formatting_sample_main_body.pdf> and

<https://journals.plos.org/plosone/s/file?id=ba62/PLOSOne_formatting_sample_title_authors_affiliations.pdf>

The manuscript meets the recommended PLOS ONE style requirements.

2. Please provide additional details regarding participant consent. In the ethics statement in the Methods and online submission information, please ensure that you have specified (1) whether consent was informed and (2) what type you obtained (for instance, written or verbal, and if verbal, how it was documented and witnessed). If your study included minors, state whether you obtained consent from parents or guardians. If the need for consent was waived by the ethics committee, please include this information.

1) Written consent was obtained from donors.

2) Donors completed a questionnaire that was signed in front of a Lifeblood staff member. No minors were involved as the minimum age for blood donation is 18 years.

In the present study we have used red blood cells and plasma for in vitro *Plasmodium falciparum* parasite cultures, which are provided by LifeBlood, Red Cross as we acknowledged in the Acknowledgements section. The blood products are de-identified and provided to us in accordance with the Material Supply Agreement with LifeBlood Red Cross, which was approved by LifeBlood Human Ethics Committee. The use of LifeBlood Red Cross blood products for in vitro cultures has also been approved by the Australian Department of Defence and Veterans Affairs (DDVA) Human Research Ethics Committee (DDVA- LREP15-014).

If you are reporting a retrospective study of medical records or archived samples, please ensure that you have discussed whether all data were fully anonymized before you accessed them and/or whether the IRB or ethics committee waived the requirement for informed consent. If patients provided informed written consent to have data from their medical records used in research, please include this information.

NA

3. Please note that PLOS ONE has specific guidelines on code sharing for submissions in which author-generated code underpins the findings in the manuscript. In these cases, all author-generated code must be made available without restrictions upon publication of the work. Please review our guidelines at <https://journals.plos.org/plosone/s/materials-and-software-sharing#loc-sharing-code>and ensure that your code is shared in a way that follows best practice and facilitates reproducibility and reuse.

We have reviewed guidelines and we will ensure our code is shared in a way that follows best practice and facilitates reproducibility and reuse

4. Thank you for stating the following financial disclosure:

“M.S-L., M.D.E. A.R.L., G.W.B., F.E.D., M.C; APP 1159384; National Health and Medical Research Council, Australia; <https://www.nhmrc.gov.au/funding>

M.S-L.; AQIRF019-2018; Advance Queensland Industry Research Fellowship; <https://advance.qld.gov.au/grants>

Australian Defence Organisation”

Please state what role the funders took in the study.  If the funders had no role, please state: "The funders had no role in study design, data collection and analysis, decision to publish, or preparation of the manuscript."

If this statement is not correct you must amend it as needed.

Please include this amended Role of Funder statement in your cover letter; we will change the online submission form on your behalf.

The amended statement is included in the cover letter.

5. We note that you have stated that you will provide repository information for your data at acceptance. Should your manuscript be accepted for publication, we will hold it until you provide the relevant accession numbers or DOIs necessary to access your data. If you wish to make changes to your Data Availability statement, please describe these changes in your cover letter and we will update your Data Availability statement to reflect the information you provide.

We will provide relevant accession numbers at acceptance.

6. We note that Figure 1 in your submission contain copyrighted images. All PLOS content is published under the Creative Commons Attribution License (CC BY 4.0), which means that the manuscript, images, and Supporting Information files will be freely available online, and any third party is permitted to access, download, copy, distribute, and use these materials in any way, even commercially, with proper attribution. For more information, see our copyright guidelines: <http://journals.plos.org/plosone/s/licenses-and-copyright>.

We require you to either (1) present written permission from the copyright holder to publish these figures specifically under the CC BY 4.0 license, or (2) remove the figures from your submission:

1. You may seek permission from the original copyright holder of Figure 1 to publish the content specifically under the CC BY 4.0 license.

We recommend that you contact the original copyright holder with the Content Permission Form (<http://journals.plos.org/plosone/s/file?id=7c09/content-permission-form.pdf>) and the following text:

“I request permission for the open-access journal PLOS ONE to publish XXX under the Creative Commons Attribution License (CCAL) CC BY 4.0 (<http://creativecommons.org/licenses/by/4.0/>). Please be aware that this license allows unrestricted use and distribution, even commercially, by third parties. Please reply and provide explicit written permission to publish XXX under a CC BY license and complete the attached form.”

Please upload the completed Content Permission Form or other proof of granted permissions as an "Other" file with your submission.

In the figure caption of the copyrighted figure, please include the following text: “Reprinted from [ref] under a CC BY license, with permission from [name of publisher], original copyright [original copyright year].”

Figure 1 was generated by our team for this Manuscript. There is no copyright information used in this Figure.

2. If you are unable to obtain permission from the original copyright holder to publish these figures under the CC BY 4.0 license or if the copyright holder’s requirements are incompatible with the CC BY 4.0 license, please either i) remove the figure or ii) supply a replacement figure that complies with the CC BY 4.0 license. Please check copyright information on all replacement figures and update the figure caption with source information. If applicable, please specify in the figure caption text when a figure is similar but not identical to the original image and is therefore for illustrative purposes only.

NA

Additional Editor Comments:

Authors need to thoroughly revise the manuscript with updated references as suggested by the reviewers. Species specificity and methodology gaps need to be properly addressed in the revised manuscript.
We have provided a statement in the discussion on why there is a difference in sensitivity of *in vivo* and *in vitro* studies at low parasitemia levels as suggested by reviewer 2. All other comments have been clarified and or addressed as suggested

[Note: HTML markup is below. Please do not edit.]

Reviewers' comments:

Reviewer's Responses to Questions

**Comments to the Author**

1. Is the manuscript technically sound, and do the data support the conclusions?

The manuscript must describe a technically sound piece of scientific research with data that supports the conclusions. Experiments must have been conducted rigorously, with appropriate controls, replication, and sample sizes. The conclusions must be drawn appropriately based on the data presented.

Reviewer #1: Yes

Reviewer #2: Yes

2. Has the statistical analysis been performed appropriately and rigorously?

Reviewer #1: Yes

Reviewer #2: N/A

3. Have the authors made all data underlying the findings in their manuscript fully available?

The [PLOS Data policy](http://www.plosone.org/static/policies.action#sharing) requires authors to make all data underlying the findings described in their manuscript fully available without restriction, with rare exception (please refer to the Data Availability Statement in the manuscript PDF file). The data should be provided as part of the manuscript or its supporting information, or deposited to a public repository. For example, in addition to summary statistics, the data points behind means, medians and variance measures should be available. If there are restrictions on publicly sharing data—e.g. participant privacy or use of data from a third party—those must be specified.

Reviewer #1: Yes

Reviewer #2: Yes

4. Is the manuscript presented in an intelligible fashion and written in standard English?

PLOS ONE does not copyedit accepted manuscripts, so the language in submitted articles must be clear, correct, and unambiguous. Any typographical or grammatical errors should be corrected at revision, so please note any specific errors here.

Reviewer #1: Yes

Reviewer #2: Yes

5. Review Comments to the Author

Please use the space provided to explain your answers to the questions above. You may also include additional comments for the author, including concerns about dual publication, research ethics, or publication ethics. (Please upload your review as an attachment if it exceeds 20,000 characters)

Reviewer #1: This research work has its own importance towards malaria diagnosis. The scientific group tried here to show the importance and utilisation of near-infrared-spectroscopy (NIRS) for diagnosis of malaria parasite (Plasmodium falciparum and P. berghei). The findings are interesting. However, the design and execution of experiments doesn’t seem adequate to establish the efficient use of NIRS for detection of malaria pathogens. The research work needs major revision before its publication. Below are few concerns observed;

We have provided response to the reviewer’s comments below
Minor Revision
Citations seems inappropriate. Many of the below citations can be omitted/replaced by predecessor relevant citations.
1. Line 87 bear citation [7-9], which seems additional as this can be cited under earlier citation [3-6]. In addition reference-7 is not related to the author’s thoughts.
2. Similarly in line 93-95 citation [12] can be cited as [11] and in [13-15] citation [13,14] are not required and reference-15 is adequate for the statement.
3. Similarly, citation [16-18] can be replaced by reference-11 in line 100-101.

We have moved these citations as suggested by the reviewer. This response addresses comments 1, 2 and 3 above (Line 91-101)

4. Line 107-108 can be rephrased as ultrasensitive quantitative PCR under citation [23] only and no need of citation [24-25]

Corrected as suggested by the reviewer (Line 109)

5. Line 110 has citation [32] not related to LAMP for malaria diagnosis, so it can be omitted.

Citation 32 has been removed (Line 111)

6. Citation [34] in line 115 is improper, however this citation is ok at other statements.

Citation 34 has been removed from this statement (Line 116)

Reviewer #2: Manuscript is well written and data are well presented. Authors have addressed an interesting and essential aspect which needs to be addressed. The need for a non-invasive methodology is necessary in malaria endemic regions for quick screening of infected and uninfected subjects. The study has pointed out that NIS can screen the parasite accurately in limited time. Using different experimental setup, the authors have also shown the efficiency of this technique. I find the article interesting and a step forward in rapid and robust screening parasite on large scale in short frame of time.
Comments:

1) If possible, the authors may include hemolyzed samples for efficiency of this methodology for detection.

We thank the reviewer for this comment which will be considered in our future study (627-629)

2) Further, authors have shown that the higher parasitemia is detected more accurately in vivo system this suggests that the lower limit of detection in vivo and in vitro is different. Authors may discuss the factors which may be responsible for this difference.

We have added a plausible reason why the lower limit of detection was lower in vivo relative to in vitro (Line 583-588)

3) It would have been more interesting to see the detection of malaria parasite in the infected individuals without any processing. The parasitemia in individual usually not high except under chronic condition or in highly endemic regions.

The results of the detection of malaria parasites in infected people have been described in our preliminary field study described by Garcia et al 2023 published by PNAS Nexus and have been cited in this work under line 144 *(Garcia, Gabriela A., et al. "Malaria absorption peaks acquired through the skin of patients with infrared light can detect patients with varying parasitemia." PNAS nexus 1.5 (2022): pgac272.)*

4) Is this methodology specifically related to Plasmodium falciparum or to other malarial parasite?

We described this methodology using *P. falciparum* and *P.berghei* samples but the technique should be assessed on other species. This statement has now been included (Line 627-629)

5) Authors assert that “specific components in biological samples” be responsible for the spectra change if true would spectra of urine from infected mice samples may also show change in spectra?

We believe that as long as there is a difference in chemical composition between samples i.e. infected and uninfected then a change in spectra would be detected. However, the difference in the chemical composition should be sufficient and not obscured by the presence of other biochemicals in the sample. We have recommended a future assessment of the technique on urine and other plasmodium samples (line 587-588)

6) Throughout the study O+ blood was used is the data. Does the detection accuracy similar in all other blood groups?
We used O+ blood group because we wanted to standardise the protocol. However, we have used the methodology on humans with different blood groups (Line 142) (Garcia et al 2022)
Authors may discuss above points throughout the manuscript. Overall, I recommend this article for publication.

We thank the reviewer for the comments and for the insights to improve our future studies and we have now included these points under the discussion section.

6. PLOS authors have the option to publish the peer review history of their article ([what does this mean?](https://journals.plos.org/plosone/s/editorial-and-peer-review-process#loc-peer-review-history)). If published, this will include your full peer review and any attached files.


If you choose “no”, your identity will remain anonymous but your review may still be made public.


**Do you want your identity to be public for this peer review?** For information about this choice, including consent withdrawal, please see our [Privacy Policy](https://www.plos.org/privacy-policy).

Reviewer #1: **Yes:** Raja Babu Singh Kushwah

Reviewer #2: No

[NOTE: If reviewer comments were submitted as an attachment file, they will be attached to this email and accessible via the submission site. Please log into your account, locate the manuscript record, and check for the action link "View Attachments". If this link does not appear, there are no attachment files.]

While revising your submission, please upload your figure files to the Preflight Analysis and Conversion Engine (PACE) digital diagnostic tool, <https://pacev2.apexcovantage.com/>. PACE helps ensure that figures meet PLOS requirements. To use PACE, you must first register as a user. Registration is free. Then, login and navigate to the UPLOAD tab, where you will find detailed instructions on how to use the tool. If you encounter any issues or have any questions when using PACE, please email PLOS at [figures@plos.org](mailto:figures@plos.org). Please note that Supporting Information files do not need this step.

*In compliance with data protection regulations, you may request that we remove your personal registration details at any time.* [*(Remove my information/details)*](https://www.editorialmanager.com/pone/login.asp?a=r)*. Please contact the publication office if you have any questions.*
